# Sport Participation and Happiness Among Veteran Footballers: The Mediating Role of Social Capital

**DOI:** 10.3390/healthcare14030396

**Published:** 2026-02-04

**Authors:** Eda Adatepe, Murat Kul, Ali Özkan, Fatih Kırkbir, Ümit Öz, Yılmaz Ünlü, Cansu Seleciler, Emre Boz

**Affiliations:** 1 Faculty of Sport Sciences, Bayburt University, 69000 Bayburt, Türkiye; edaadatepe@bayburt.edu.tr (E.A.); muratkul@bayburt.edu.tr (M.K.); 2 Faculty of Sport Sciences, Yozgat Bozok University, 66000 Yozgat, Türkiye; ali.ozkan@bozok.edu.tr; 3 Faculty of Health Sciences, Karadeniz Technical University, 61000 Trabzon, Türkiye; fatihkirkbir@ktu.edu.tr; 4 Health Services Vocational School, Bayburt University, 69000 Bayburt, Türkiye; umitoz@bayburt.edu.tr; 5 Faculty of Sport Sciences, Bolu Abant İzzet Baysal University, 14000 Bolu, Türkiye; yilmaz.unlu@ibu.edu.tr; 6 Graduate Education Institute, Bayburt University, 69000 Bayburt, Türkiye; cansuseleciler@gmail.com

**Keywords:** veteran footballers, sport participation, happiness, social capital, aging

## Abstract

**Aim:** As the global population is aging rapidly, promoting physical activity in later life is increasingly seen as a good strategy to enhance and sustain the social and psychological well-being of older adults from a positive aging perspective. This study explored how social capital influences the relationship between playing football and happiness in veteran footballers. Specifically, it aimed to understand if social capital acts as a mediator between these two factors. **Materials and Methods:** This cross-sectional study was performed on a convenience sample of 423 veteran footballers aged from 38 to 59 years who completed a survey at 35th Sakarya Veterans Football Tournament in Sakarya in north-west Turkey. Football participation was assessed using the Serious Leisure Inventory. Social capital was assessed in both cognitive and structural aspects. A single-item scale assessed general happiness. Data were analyzed with Pearson correlation coefficients and were calculated using SPSS (version 24) to assess the direction and strength of the links between the investigated variables. Path coefficients were calculated through regression analyses. For testing mediation effects, the study utilized Hayes’ bootstrapping method, executed with the Version 4.2 Beta of the PROCESS macro. **Results:** According to the research findings, the direct effect of football participation on happiness was determined to be 0.43 (*p* < 0.01). While community involvement played a significant mediating role with a coefficient of 0.11 (95% CI [0.05, 0.15]), the social trust and neighborhood dimensions of social capital did not yield statistically significant effects. Collectively, the model explains 24% of the variance in happiness (*R*^2^ = 0.24), with a total effect of football participation calculated at 0.57 (*p* = 0.000). **Conclusions:** it is believed that social environments that include participation in sport can contribute to successful and comfortable aging by greatly enhancing the overall well-being and happiness of older athletes/adults.

## 1. Introduction

The phenomenon of population aging is increasingly recognized as a significant global trend, with projections indicating that individuals aged 60 years and older will surpass the number of children under five years old. This shift highlights the rapid pace of demographic changes occurring worldwide. According to the World Health Organization, the global population aged 60 and above is expected to reach 2.1 billion by 2050, which will represent a substantial increase from 1 billion in 2019 [1]. This demographic transition is not uniform across regions; for instance, countries in East Asia are experiencing particularly rapid aging compared to some European nations, where the aging process is slower [2]. Successful aging is a multifaceted concept that encompasses various dimensions of health, well-being, and social engagement as individuals grow older. The definition of successful aging has evolved over time, reflecting a shift from merely the absence of disease to a more holistic view that includes psychological, social, and occupational factors. Rowe and Kahn’s model of successful aging emphasizes three key components: low probability of disease and disability, high cognitive and physical functioning, and active engagement with life [3].

The perspective of promoting later life as a period of enjoyment, growth, creativity, independence [4], and development has gained traction in recent research, moving beyond traditional views that emphasize loneliness, disengagement, and decline. This shift is reflected in various studies that advocate for a more positive and proactive approach to aging [5]. This body of positive aging literature has driven a global health promotion movement, involving governments, non-profit organizations, and businesses. It highlights a cultural focus on sport, physical activity, exercise, recreation, and leisure as key strategies for enhancing and maintaining the social and psychological well-being of older adults [6].

Happiness is a complex construct that has been extensively studied across various disciplines, including psychology, sociology, and economics. It is often conceptualized as a subjective evaluation of one’s life circumstances, which includes both immediate emotional experiences and broader cognitive assessments. Ref. [7] defines happiness in terms of subjective well-being, which encompasses life satisfaction, the presence of positive moods, and the absence of negative moods. This definition emphasizes the importance of individual perceptions and experiences in assessing happiness. Ref. [8] expands on the cognitive aspects of happiness by discussing how individuals assess their life circumstances. This appraisal process involves comparing one’s current situation to personal standards or expectations, which can significantly influence feelings of happiness and life satisfaction.

Happiness, closely linked to an individual’s health and quality of life [9], has attracted significant interest from researchers studying various populations. The literature describes the relationship between aging and happiness as a U-shaped relationship among older adults [10], after controlling for demographic variables [11]. It means that happiness tends to be higher in youth, dips during middle age [12], and rises again in older adulthood. Ref. [11] discusses the complexity of the happiness–age relationship, noting that while many researchers report a U-shape, the underlying factors contributing to this pattern are not fully understood.

Social epidemiology is a field that includes the social context in explaining a person’s health status. From this context, scholarly research increasingly acknowledges that social factors, including structural characteristics and social behaviors or experiences, significantly impact various psychological conditions and mental health results. Such consequences encompass self-efficacy, self-worth, depression scales, emotional management, and holistic well-being [13]. Social capital is being progressively acknowledged as a vital determinant of general life satisfaction among diverse demographic groups. This concept encompasses the networks, relationships, and norms that facilitate cooperation and social cohesion, which in turn influence individual well-being. Numerous studies have explored the intricate relationship between social capital and happiness, revealing that social interactions, trust, and community engagement significantly contribute to overall life satisfaction [14,15,16,17,18].

Although there are different definitions of social capital in various fields, it is known that social capital is accepted as a fundamental characteristic of social relations in the field of social sciences. In the narrowest sense, social capital is the tool used to maximize the benefits that can be obtained through cooperation and participation and to achieve the goal in social relations [19]. An alternative conceptualization of social capital involves the capacity to obtain actual or prospective assets by participating in social frameworks and interconnected networks [20]. Significant studies argue that social capital is an important source of mental well-being and general findings of research have indicated that social capital was a predictor of happiness [2,21,22].

Compared to individual physical activity or exercise, participating in sports is often a more effective way to promote positive social and psychological outcomes, largely because of its inherently social nature [23]. However, significant gaps remain in the existing scholarship regarding the consequences of athletic involvement in middle-aged and elderly cohorts. Initially, numerous earlier investigations have chiefly concentrated on the influences of bodily movement or training. Second, while numerous studies have examined the status and effects of sport participation in children and adolescents, relatively few have empirically explored its effects on veteran athletes. Lastly, the current body of research presents inconsistent and fragmented findings, largely attributable to the limited number of empirical studies. To address these gaps, the primary aim of this study is to investigate the influence of sport participation—specifically football—on individual happiness, with particular attention to the mediating role of social capital among veteran footballers.

Participation in various sports, including football, has been increasingly reported among older adults, reflecting a shift towards recognizing the benefits of physical activity in later life. This trend is supported by a growing body of literature that emphasizes the positive impacts of sports on health, social engagement, and overall well-being for older populations [24,25,26]. In their study titled “A research on the question of why football comes to mind first when it comes to sports today?”, the authors state that football will continue to exist as a social phenomenon that will always increase its popularity with its sociological and psychological dimensions that include the social structure of families that appeal to the masses.

Veteran football and leagues are as popular as football in Turkey [27,28]. Veteran football associations have missions such as uniting the resources and strengths of the master and veteran sports clubs operating in Turkey, pioneering the sports of our individuals who are forty years of age or older who are devoted to football, and providing the necessary contributions in line with the needs of our people who want to do sports as healthier individuals. In addition, there are various associations and leagues, with the idea of social responsibility, in order to take our people away from the intensity and tempo of work, to carry the unity, dialog, and human relations within the family to a more solid foundation, to set an example for all people by explaining the benefits of sports to our young people and children, and to provide the necessary interest and support. These are as follows:

- Turkish Masters and Veterans Football League (TMVFL): one of the best known and most comprehensive veteran football leagues in Turkey.
- Istanbul Veterans League: based in Istanbul, this league is an organization for former football players and amateur veteran football players in Istanbul.
- Aegean Veterans League: Based in and around Izmir, this league is organized for veteran football players in the Aegean Region. Both friendly matches between local teams and tournaments are organized.
- Mediterranean Veterans League: Organized in cities such as Antalya, Mersin, and Adana, this league is a platform for veteran footballers in the Mediterranean Region to come together. It offers football excitement to the people of the region with tournaments and special matches.
- Ankara Veterans League: organized in the capital city of Ankara, this league is an organization where veteran teams consisting of former football players compete.

In the Turkish context, while veteran sports participation has historically been dominated by male-centric football structures [29], there is an emerging, albeit less institutionalized, trend of female veteran sports involvement. Unlike the highly organized male veteran football leagues such as the TMVFL, female veteran participation is more prominently formalized in other disciplines, most notably volleyball. The Turkish Volleyball Federation (TVF) officially sanctions annual veteran championships, where women’s teams compete in age categories from +35 to +50, demonstrating a well-established institutional framework for aging female athletes [30]. In football, however, female veteran participation remains largely informal or limited to symbolic matches in international master tournaments, reflecting a significant gender gap in the formalization of social networks through sport [31]. This gender-based disparity in structural social capital suggests that while men access ‘bridging’ capital through veteran football, women often rely on other disciplines or informal groups to achieve similar psychological outcomes in later life.

This study extends the Serious Leisure Perspective (SLP) by integrating it with a multidimensional social capital model. While the SLP posits that the systematic pursuit of an activity leads to social benefits [32,33], the findings reveal that these benefits are not uniform, thereby developing the existing theoretical framework. It has been found that the mediating effect of social capital is specific to community-based structural participation, thus providing a more nuanced understanding of how leisure activities requiring high commitment (e.g., veteran football) transform social resources into subjective well-being [34,35]. In the case of aging athletes, it has been observed that “bridging” social capital, crystallized in organized tournaments, can take precedence over “bonding” or informal neighborhood ties in promoting happiness [36]. This situation demonstrates that formalizing social networks through sport for the aging population is a fundamental driver of mental health [37] and represents a distinction that clarifies the limits of social capital’s impact on happiness [38,39].

### 1.1. Research Gap

Although the positive correlation between sports participation and happiness is well documented in the general population, the underlying social mechanisms—particularly how different dimensions of social capital act as catalysts—have not been sufficiently researched in the context of aging athletes. The current literature generally treats physical activity as a general health behavior, but research focusing on middle-aged adults’ participation in “serious leisure activities” is significantly lacking. This study addresses these gaps by shifting the focus from everyday physical activity to sports participation requiring high commitment, such as veteran football. By separating social capital into structural and cognitive dimensions, our research determines the precise mediating role of “community participation” over other social factors. Ultimately, this study sought to show whether all social interactions provide equal psychological benefits and that it is the organized and commitment-demanding nature of veteran sports that transforms participation into happiness. Thus, the findings offer a more nuanced theoretical framework for understanding successful aging through specific social environments.

### 1.2. Hypotheses Development

While there is no study examining the relationship between sports participation and happiness among veteran footballers, studies on sports participation among middle-aged and older adults have found positive effects of sports on happiness. Ref. [40] emphasizes that sports participation has a positive effect on the subjective well-being of the population, which is further enhanced by social interaction. Ref. [41] explored how football affects the well-being of male asylum seekers living in a UK hotel. The study involved giving participants the chance to travel and play football twice a week for seven weeks. The results showed notable improvements in their well-being, including enhanced life satisfaction, a greater sense of the value of their activities, and increased happiness. In addition, [22], in their research titled Small Touches to Happiness in an Aging World, stated that the happiness levels of the elderly who play petanque sports are high. According to the findings of [21], it was determined that 10-week recreational activities reduced the perceived stress levels of middle-aged and older adult women by 33.57%, while increasing their happiness levels by 20.58% and life satisfaction levels by 14.39%. Hence, the following hypothesis was established:

**Hypothesis 1** **(H1):** *Football Participation to General Happiness.*

**Hypothesis 2** **(H2):** *Football Participation to Community Participation.*

**Hypothesis 3** **(H3):** *Football Participation to Feelings of Trust and Safety.*

**Hypothesis 4** **(H4):** *Football Participation to Neighborhood Connections.*

**Hypothesis 5** **(H5):** *Community Participation to General Happiness.*

**Hypothesis 6** **(H6):** *Feelings of Trust and Safety to General Happiness.*

**Hypothesis 7** **(H7):** *Neighborhood Connections to General Happiness.*

Hypotheses 8, 9, and 10 describe the mediating roles. These are the indirect pathways from football participation to general happiness, operating through the mediating variables.

**Hypothesis 8** **(H8):** *Football Participation to Community Participation (Mediating Variable M_1_) to General Happiness.*

**Hypothesis 9** **(H9):** *Football Participation to Feelings of Trust and Safety (Mediating Variable M_2_) to General Happiness.*

**Hypothesis 10** **(H10):** *Football Participation to Neighborhood Connections (Mediating Variable M_3_) to General Happiness.*

## 2. Materials and Methods

### 2.1. Research Method

This study employed a quantitative, cross-sectional survey design to examine the relationships between football participation, social capital, and happiness among veteran footballers. A convenience sampling method was used to recruit participants who were readily accessible. Data were collected using structured questionnaires and analyzed using statistical mediation analysis to determine whether dimensions of social capital mediated the relationship between football participation and general happiness.

### 2.2. Research Model/Design

This study adopted a cross-sectional research design, meaning that data was collected from participants at one specific point in time rather than over a longer period. The purpose was to assess the relationships between football participation, social capital, and happiness among veteran footballers based on their current experiences and perceptions. Participants were surveyed only once, and there was no follow-up or longitudinal tracking. This design is appropriate for identifying associations between variables within a population at a given moment.

### 2.3. Participants

A convenience sampling strategy was employed to recruit participants from the 35th Sakarya Veterans Football Tournament, an event recognized by UEFA as a premier special football tournament in Turkey. The study population consisted of 599 registered veteran footballers across 79 teams, categorized into three distinct age groups: youth veteran (34–39), intermediate veteran (42–48), and veteran (49+). To ensure a representative cross-section of the tournament, researchers were present during the group stages and invited players from all three categories to participate. Of the 599 registered players, 443 (74%) voluntarily agreed to participate in the paper-and-pencil survey. To mitigate potential social desirability bias, participants were provided with a private space to complete the survey, assured of total anonymity, and informed that their responses would have no impact on their tournament standing. Following a screening for data integrity, 20 incomplete surveys were excluded, resulting in a final analytical sample of 423 male participants (M_age_ = 45.84, SD = 5.41). The sample size (n = 423) provides a high response rate (70.6% of the total registered population), which enhances the robustness of the findings within the context of veteran football. Among the sample, 66% were aged between 38 and 47, while 34% were between 48 and 59. In terms of education, 43.7% held a Bachelor’s degree, and 26.5% were high school graduates. Regarding skill level, 48% of participants rated themselves as ‘good players’ (a rating of 3 on a scale from 1 to 4), where 1 indicates poor skill and 4 indicates very good skill. More detailed demographic characteristics of the respondents are presented in Table 1.

### 2.4. Ethical Considerations

This study was conducted in strict accordance with the principles of the Declaration of Helsinki. Ethical approval was granted by the Ethics Committee of Bayburt University (Date: 6 October 2023, Decision No: 284; Permission No: E-15604681-100-159575). Prior to data collection, all participants were provided with an Informed Consent Form that detailed the study’s purpose, the types of questions to be asked, and the estimated time for completion. It was explicitly stated that participation was entirely voluntary and that participants could withdraw from the survey at any point without providing a reason. To ensure data confidentiality and anonymity, no personally identifiable information (such as names or ID numbers) was collected. All physical survey forms were stored in a secure location accessible only to the research team, and digital data were kept on password-protected devices to prevent unauthorized access.

### 2.5. Data Collection Tools

#### 2.5.1. Football Participation

To assess the degree of soccer participation, six items from the Serious Leisure Inventory Measures (SLIM) were employed, as this instrument is frequently applied and substantiated by previous authors [42]. In 2016, Ref. [43] adapted the 9-point Likert-type scale consisting of 18 dimensions and 54 items into Turkish with the study titled “Serious Leisure Inventory and Measurement: Validity and reliability analyses”. Participants evaluated their dedication and engagement levels regarding football on a 9-point Likert scale, spanning from 1 (strongly disagree) to 9 (strongly agree). Elevated values reflected a deeper commitment to football as a serious leisure activity. The primary attributes of serious leisure were captured through six distinct sub-factors: perseverance, career progression, career advancement, effort, unique ethos, and identification. Illustrative examples from the instrument include ‘I persist until I conquer challenges encountered in football’ (perseverance), ‘I train to enhance my footballing abilities’ (effort), and ‘I embrace the core values of my soccer collective’ (unique ethos). Following the approach of [42], this metric functioned as a summative index to demonstrate fluctuations in seriousness intensity. An average was calculated across the six dimensions (M = 7.70, SD = 0.989), while overall internal consistency was confirmed via Cronbach’s alpha (α = 0.936).

#### 2.5.2. Social Capital

The scale developed by [44] consisting of 14 items and 6 factors was used to measure perceived social capital. Adopted sub-dimensions assessed both cognitive (i.e., feelings of trust and safety) and structural social capital (i.e., community participation, neighborhood connections). The tool was designed to assess three core constructs, each measured by three items. The example items include “Are you an active member of a local organization or club (e.g., sport, craft, social club)?” (Community participation), “Do you feel safe walking down your street after dark” or “Do you agree that most people can be trusted?” (Feelings of trust and safety), and “If you were caring for a child and needed to go out for a while, would you ask a neighbor for help?” (Neighborhood connections and reciprocity). Each item was assessed with a 4-point Likert-type scale ranging from 1 (no, not at all) to 4 (yes, very much). The Cronbach’s alpha values were 0.716 (community participation), 0.691 (feelings of trust and safety), and 0.745 (neighborhood connections and reciprocity), respectively, indicating acceptable consistency [45].

#### 2.5.3. Happiness

The single-item scale developed and validated by [46] was utilized to assess overall perceptions of well-being. Answers to the inquiry “Do you feel happy in general” were distributed on a spectrum from 1 (lowest) to 10 (highest). Greater values represent an increased degree of joy. Consistent with prior research, because a single-faceted concept (namely, general happiness) was evaluated, this one-item measurement was considered suitable [47,48,49,50]. In this research, the single-item general feelings of happiness scale average and standard deviation are M = 8.11, SD = 1.834.

### 2.6. Covariates

The analysis accounted for age, sex, education, and football expertise as control parameters, given that such demographic indicators are known to be linked with well-being and social resources. Despite the lack of a clear agreement on how social capital fluctuates over one’s life cycle, the scholarship highlights that there are meaningful connections between age and social capital, which can be positive, negative, or neutral [51,52,53]. The sample of this study consists entirely of male participants. When the studies are examined, men’s average social capital in some studies and women’s average social capital in some studies differed. Again, there were studies in which there was no differentiation according to gender [54,55,56,57]. For education level, past research has shown that education is strongly and positively correlated with individual social capital [58,59,60,61]. In many studies, happiness has been affected by age [62,63,64], education level [65,66], and gender [67,68] variables and statistically significant relationships have been found in these studies. In another study, it was shown that there was a significant relationship between emotional aspects and functional motor skills of football players [69].

### 2.7. Statistical Analyses

Pearson correlation coefficients were computed using SPSS (version 24) to “examine the direction and strength of relationships between the research variables”. Regression analyses were conducted to estimate the path coefficients. In each analysis, path a represents the relationship between the predictor variable, Football Participation (FP), and the three subdimensions of the mediating variable, Social Capital (SC). Path b links the subdimensions of SC—SC-Community, SC-Trust and Safety, and SC-Neighborhood—to the outcome variable, Happiness. Path c’ connects FP with Happiness while accounting for the mediating role of SC, and path c reflects the total effect of FP on Happiness. The indirect effect was calculated as the product of the a and b path coefficients.

To evaluate the indirect pathways, we utilized the bootstrapping technique proposed by Hayes [70] through the PROCESS macro (Version 4.2 Beta) [71]. This procedure generates 5000 bootstrap resamples for bias adjustment [72,73] to establish the 95% confidence intervals (CIs). When a 95% CI excludes zero, it confirms that the indirect effects [74,75] are statistically significant at the *p* < 0.05 level, indicating a departure from zero.

## 3. Results

Table 2 presents descriptive statistics and Pearson correlation coefficients of the study variables. Football participation was significantly positively associated with two subdimensions of social capital (r = 0.269, *p* < 0.01; r = 0.083, *p* > 0.01; r = 0.241, *p* < 0.01) and general happiness (r = 0.380, *p* < 0.01). All three subdimensions of social capital were significantly positively associated with general happiness (r = 0.384, *p* < 0.01; r = 0.137, *p* < 0.01; r = 0.313, *p* < 0.01).

The outcomes of the regression model demonstrated that subjective well-being was remarkably influenced by engagement in football (adjusted *R*^2^ = 0.227, F [4,418] = 32.009, *p* < 0.001). In addition, general happiness was significantly predicted by community participation (adjusted *R*^2^ = 0.146, F [4,418] = 72.918, *p* < 0.001). Therefore, as seen in these analyses, hypotheses 1, 2, 4, 5, 6, and 7 were supported, and only hypothesis 3 was not supported.

According to Table 3, Football Participation predicts SC-Com (M1) and SC-Nei (M3) mediator variables (b = 0.40 and b = 0.35, respectively). Football participation explains 7% of the variance in the SC-Com variable and 6% of the variance in the SC-Nei variable. Happiness, which is the output variable, is statistically significantly predicted by Football Participation (b = 0.44) and SC-Com (b = 0.25). FP and SC-Com explain 24% of the variability in the output variable Happiness. Table 4 presents the direct and indirect effects of Football Participation on Happiness.

The mediation analysis results indicated that the total effect of football participation on happiness was significant (β = 0.44, t = 6.18, *p* < 0.001). When examining the indirect effects through the three dimensions of social capital, only Community Participation (M1) was found to be a significant mediator (Indirect Effect = 0.10, SE = 0.03, 95% CI [0.05, 0.16]). In contrast, the indirect effects of Trust and Safety (M2) (Indirect Effect = 0.001, 95% CI [−0.01, 0.02]) and Neighborhood Connections (M3) (Indirect Effect = 0.03, 95% CI [−0.01, 0.07]) were not significant, as their confidence intervals included zero.

When Table 4 is analyzed, the result shows that the total effect of football participation on happiness is 0.57. This total effect is realized through the direct effect of Football Participation and the partial mediation of the SC-Com variable. When the mediation effects are analyzed, it can be said that football participation indirectly affects happiness through SC-Com and this indirect effect is statistically significant (β = 0.07, *p* < 0.01). When the indirect effects were analyzed statistically, it was observed that the SC-Com variable had a statistically significant indirect effect. In addition, approximately 20% of the total effect of Football Participation on Happiness is due to the SC-Com variable (0.11/0.57). When the mediator variable is considered, 20% of the total effect is due to the indirect effect. According to Table 4, hypothesis 8 was supported, whereas hypotheses 9 and 10 were not supported.

**Figure 1:**
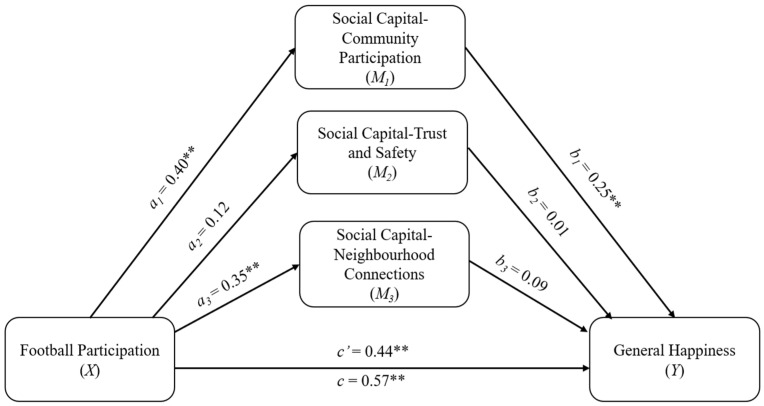
Path analysis. Notes: ** *p* < 0.01.

The mediating effect of three social capital dimensions in the relationship between football participation and general happiness is shown below. All presented effects are unstandardized; a_1,_ a_2_, and a_3_ are effects of football participation on social capital dimensions; b_1_, b_2_, and b_3_ are effects of social capital dimensions on general happiness; c′ is the direct effect of football participation on general happiness; c is the total effect of football participation on general happiness (Figure 1).

## 4. Discussion

This investigation primarily sought to understand the intervening influence of social capital on the association between sports involvement and life satisfaction among senior footballers. Specifically, we explored how football participation indirectly affects happiness through community engagement, trust and safety levels, and neighborhood networks, while accounting for background variables like age, educational level, and football expertise (i.e., age, education, football skills).

The findings suggested that football participation was significantly and positively predicted by general happiness among veteran footballers. This finding supports previous research on the positive psychological outcomes of sports participation. Football participation has a significant impact on individuals’ happiness levels. Football is one of the most popular sports with approximately 250 million people actively participating and attracting 1.4 billion people worldwide [76]. This wide participation shows the potential of soccer as a social activity to improve the quality of life of individuals. Research reveals that soccer not only provides physical activity, but also plays an important role in terms of social interaction and community building. Recreational football participants consider this activity as an opportunity to socialize and this positively affects their happiness levels [77].

This investigation diverges from prior scholarship by analyzing how social capital serves as a mediator between athletic involvement and overall well-being. While numerous earlier works have assessed the impact of sporting activity on social or psychological results, the current research evaluated the prospective connection between social resources and happiness through a social epidemiology lens. Our data indicate that the Community Participation component of social capital plays a vital and positive intervening role in the link between football engagement and general happiness in senior players. Two subdimensions (i.e., feelings of trust and safety and neighborhood connections) of social capital were not significant predictors of happiness nor significant mediators. Research indicates that social capital, particularly through active participation in social activities, correlates positively with individual happiness, emphasizing the importance of social engagement as a mediator in this relationship [14]. Similarly, those social activities associated with physical activities can enhance social networks, which in turn contribute to increased happiness [78]. This suggests that community participation is a critical factor in fostering happiness, aligning with the findings of veteran footballers where participation in the sport enhances social connections and overall well-being. Conversely, the study by found that while feelings of safety and trust are often considered important predictors of happiness, they did not consistently demonstrate significant effects in their analysis [79]. This aligns with the assertion that not all dimensions of social capital are equally influential in mediating happiness. Furthermore, research supports the notion that social participation, rather than trust or neighborhood connections, is a more robust predictor of happiness among adults [80]. This is consistent with the findings that suggest community participation is the key driver of happiness in veteran footballers, while other forms of social capital, such as trust and safety, do not hold the same weight.

The data for this study were obtained at a sport event. The finding aligns with earlier research indicating that participating in sports events can positively impact psychological well-being. The positive relationship between sport event participation and psychological well-being has been well-documented in various studies. Participation in sports is associated with numerous mental health benefits, including reduced symptoms of depression and anxiety, as well as improved overall psychological well-being. Sivaramakrishnan et al. [39] found in their study titled “Psychosocial outcomes of sport participation for middle-aged and older adults: a systematic review and meta-analysis” that participation in sports among middle-aged and older adults is associated with numerous psychosocial benefits that remain consistent regardless of sport-specific characteristics. In addition, numerous studies support these findings [81,82,83,84]. However, there is a lack of empirical studies examining the link between a sense of community through participation in a sport and various psychological health outcomes as a result. If we want to clarify the relationship between sense of community and sport in middle-aged and older adults, it may be necessary to distinguish between participation in sport and participation in a sport event.

Our findings indicate that sport participation can serve as an effective multilevel social capital intervention for improving health among older adults and underscore the significant need for future research on sport-based social capital interventions. Participation in local-based sport programs and leagues has been shown to significantly enhance neighborhood networks and support systems. These programs serve as a vital platform for community engagement, fostering social connections among participants. Research indicates that informal sport participation can effectively reach diverse communities, promoting social inclusion and physical activity, which are essential for public health objectives [85].

The positive impact of sport participation on social capital seems apparent, as sport provides an effective platform for individuals to socialize and expand their social networks and relationships. On the other hand, some scholars have emphasized the existence of a dark side to the concept of social capital [86]. While social capital may represent trust, social networks, beliefs, and norms for some, it can also signify opportunities for exploitation, misuse, and manipulation for others. This should be considered in sport-based interventions aimed at enhancing social capital.

In this study, we used the instrument for serious leisure to measure the level of sport participation among veteran footballers. The concept of serious leisure has been increasingly recognized as a significant framework for understanding the participation of older adults in sports and leisure activities. This construct highlights the importance of sustained engagement in leisure activities that require specialized skills and knowledge, which can lead to enhanced life satisfaction and well-being among older individuals. Most of the participants in our study were serious sports participants (M = 7.70 (out of 9), SD = 0.989). For instance, Stebbins’ foundational work on serious leisure emphasizes that this form of leisure involves a commitment to activities that provide deep satisfaction and often require significant effort and skill [87]. This framework has been applied in various contexts, including sports participation among older adults, where serious leisure activities are linked to improved health outcomes and social connections [34]. The findings further support this notion, demonstrating that serious involvement in leisure activities contributes positively to happiness and successful aging among older adults, particularly those engaged in sports clubs [88,89].

The findings of this study offer a more nuanced theoretical understanding of how sport participation facilitates well-being in later life. From a Sport Psychology perspective, the significant mediating role of community participation can be explained through Self-Determination Theory (SDT). Specifically, veteran football provides a structured environment that fulfills the fundamental psychological need for relatedness. Unlike informal physical activities, the ‘social world’ of a football tournament creates a unique identity and sense of belonging that are crucial for psychological resilience during the aging process. Furthermore, when interpreted through the SLP, our results suggest that the happiness of veteran athletes is not a product of general social interactions, but rather of their high-commitment involvement in a specialized sport community. This distinction explains why ‘community participation’ was a significant mediator while ‘neighborhood connections’ were not. This suggests a refinement of Social Capital Theory in the context of aging: for this population, bridging capital (formed through shared athletic interests) appears to be more psychologically rewarding than bonding capital (localized residential ties). Consequently, organized sport acts as a ‘social vehicle’ for successful aging, compensating for the shrinking social networks often associated with retirement and later life stages.

A notable finding of this study is that while community participation acted as a strong mediator, trust and neighborhood connections did not show significant mediating effects. Theoretically, this distinction can be explained through the SLP. For veteran footballers, the psychological benefits and happiness are primarily derived from their structural involvement and active role within the specific ‘social world’ of the football community, rather than generalized social trust or localized neighborhood ties. This suggests that in the context of successful aging through sports, the functional and organized nature of the athletic community provides a more direct pathway to well-being than informal or cognitive social bonds.

## 5. Study Limitations

Despite its contributions, this study has several limitations that offer directions for future research. First, the cross-sectional and correlational design precludes the establishment of definitive causal inferences. While our theoretical framework posits that football participation enhances happiness, the temporal ordering remains unverified; thus, the potential for reverse causality—where inherently happier individuals are more likely to engage in veteran sports—cannot be entirely dismissed. Future research employing longitudinal or mixed-method approaches is encouraged to provide more robust evidence regarding these causal pathways.

Second, the sample was limited to male veteran footballers from a single geographical region (Sakarya, Turkey). Given that gender and cultural context significantly influence the formation of social capital and its impact on well-being, the external validity of these findings remains constrained. To enhance generalizability, future studies should adopt comparative designs that include female veteran athletes and more geographically diverse populations. Such inclusivity would provide a nuanced understanding of gender-specific patterns and regional differences in the relationship between sports participation and psychological health.

Third, this research focused exclusively on football. Future investigations could broaden the scope by comparing the psychological benefits across various team and individual sports, particularly among middle-aged and older adults. Finally, while this study emphasized social capital as a mediator, future research should incorporate a wider range of socio-demographic and environmental variables. Such comprehensive analyses would further clarify the complex interplay between sports engagement, social dynamics, and subjective well-being in the aging population. It is also believed that conducting these studies using different research models (e.g., experimental research models) in future research will yield further findings related to the subject.

## 6. Conclusions and Recommendations

This study significantly advances the academic discourse regarding the intricate relationship between consistent sports participation and the subjective well-being of veteran footballers, with a particular emphasis on the crucial mediating role played by social capital. Through the application of rigorous regression analyses, the research establishes that both active involvement in sports and the structural components of social capital—most notably community engagement—act as powerful and significant predictors of an individual’s overall happiness.

The findings suggest that the act of playing football is far more than a mere physical exercise; it serves as a vital platform for community participation that fundamentally bolsters the daily psychological resilience and mental health of aging athletes. By fostering these deep-seated social bonds and maintaining physical vigor, such sporting activities provide a robust framework for achieving successful, high-quality, and active aging. Looking forward, this research opens new avenues for scholarly inquiry, suggesting that future studies should more deeply investigate how various sporting contexts influence the density of social connections and the breadth of community engagement. Furthermore, there is a clear need to develop and implement targeted organizational strategies designed to maximize the inherent social dividends of sports, ultimately enhancing the general well-being and life satisfaction of individuals as they age.

The findings of this study offer critical insights for sport policies and active aging programs, suggesting a strategic shift from promoting informal physical activities toward fostering organized, high-commitment sports communities. Since our results identify structural community participation as the primary driver of happiness, policymakers should prioritize funding for veteran leagues and inter-municipal tournaments that provide a sense of formal membership and shared social identity. Sport organizations are encouraged to leverage the ‘Serious Leisure’ perspective by designing programs that offer veteran athletes opportunities for skill development, leadership roles (e.g., team captaincy), and regular competitive cycles, which collectively enhance psychological well-being. Furthermore, local governments should expand events like the Sakarya Veterans Tournament, transforming them into social festivals that facilitate ‘bridging social capital’ and broad community engagement among the middle-aged and older populations. Finally, active aging infrastructure should integrate mental health awareness within sport clubs, training administrators to recognize that the maintenance of robust social networks is as vital as physical fitness in ensuring successful aging.

## Figures and Tables

**Table 1:** Demographic characteristics of the study participants (n = 423).

Characteristics	Groups	n	%
Age	38–42	146	34.5
	43–47	134	31.6
	48–52	76	17.9
	53–59	67	15.8
Education	Middle School	34	8.0
	High School	112	26.5
	Vocational School	30	7.1
	Bachelor’s Degree	185	43.7
	Master	52	12.3
	Doctorate (PhD)	10	2.4
Football Skill Rating	(1) Bad	14	3.3
	(2) Moderate	115	27.2
	(3) Good	200	47.3
	(4) Very good	94	22.2

**Table 2:** Descriptive statistics and Pearson correlation coefficients of study variables.

Variable	1	2	3	4	5	Mean (SD)
1. FP	1	0.269 **	0.083	0.241 **	0.380 **	7.70 (0.989)
2. SC-Com		1	0.310 **	0.624 **	0.384 **	2.85 (0.570)
3. SC-Trust			1	0.280 **	0.137 **	2.57 (0.641)
4. SC-Nei				1	0.313 **	2.92 (0.523)
5. Happiness					1	8.11 (1.834)

FP—Football Participation, SC-Com social capital—community participation, SC-Trust social capital—feelings of trust and safety, SC-Nei social capital—neighborhood connections. ** *p* < 0.01.

**Table 3:** Unstandardized coefficients and 95% bootstrap confidence intervals.

	M_1_			M_2_			M_3_			Happiness (Y)		
	Estimation	LLCI	ULCI	Estimation	LLCI	ULCI	Estimation	LLCI	ULCI	Estimation	LLCI	ULCI
FP (X)	0.40 **	0.26	0.53	0.12	−0.18	0.25	0.35 **	0.21	0.48	0.44 **	0.30	0.57
SC-Com (M_1_)										0.25 **	0.14	0.36
SC-Trust (M_2_)										0.01	−0.08	0.10
SC-Nei (M_3_)										0.09	−0.02	0.19
	R^2^ = 0.07			R^2^ = 0.007			R^2^ = 0.06			R^2^ = 0.24		

** *p* < 0.01, LLCI: Lower limit confidence interval; ULCI: Upper limit confidence interval.

**Table 4:** Direct and indirect effects of football participation on happiness.

	Unstandardized			Standardized Coefficients	
	Estimation	LLCI	ULCI		*p*-Value
Direct effect	0.43 **	0.30	0.57	0.29	0.000
Indirect effect on M_1_	0.11 **	0.05	0.15	0.07	0.001
Indirect effect on M_2_	0.01	−0.01	0.01	0.00	0.843
Indirect effect on M_3_	0.02	−0.02	0.06	0.02	0.412
Total effects	0.57 **	0.43	0.70	0.38	0.000

** *p* < 0.01, M_1_: Social Capital-Community, M_2_: Social Capital-Trust-Safety, M_3_: Social Capital-Neighborhood connections.

## Data Availability

The datasets are available from the corresponding author on request.
